# A novel conjugative transposon carrying an autonomously amplified plasmid

**DOI:** 10.1128/mbio.02787-23

**Published:** 2024-01-23

**Authors:** Joseph H. Vineis, William S. Reznikoff, Dionysios A. Antonopoulos, Jason Koval, Eugene Chang, Bailey R. Fallon, Blair G. Paul, Hilary G. Morrison, Mitchell L. Sogin

**Affiliations:** 1 Josephine Bay Paul Center, Marine Biological Laboratory, Woods Hole, Massachusetts, USA; 2 Biosciences Division, Argonne National Laboratory, Argonne, Illinois, USA; 3 Section of Gastroenterology, Department of Medicine, Knapp Center for Biomedical Discovery, The University of Chicago, Chicago, Illinois, USA; University of Maryland School of Medicine, Baltimore, Maryland, USA

**Keywords:** conjugal transposon, *Bacteroides fragilis*, microbial evolution, host–microbe interactions, CTnDOT, antibiotic resistance

## Abstract

Tetracyclines serve as broad-spectrum antibiotics to treat bacterial infections. The discovery of new tetracycline resistance genes has led to new questions about the underlying mechanisms of resistance, gene transfer, and their relevance to human health. We tracked changes in the abundance of a 55-kbp conjugative transposon (CTn214) carrying *tetQ,* a tetracycline resistance gene, within a *Bacteroides fragilis* metagenome-assembled genome derived from shotgun sequencing of microbial DNA extracted from the ileal pouch of a patient with ulcerative colitis. The mapping of metagenomic reads to CTn214 revealed the multi-copy nature of a 17,044-nt region containing *tetQ* in samples collected during inflammation and uninflamed visits. *B. fragilis* cultivars isolated from the same patient during periods of inflammation harbored CTn214 integrated into the chromosome or both a circular, multi-copy, extrachromosomal region of the CTn214 containing *tetQ* and the corresponding integrated form. The tetracycline-dependent mechanism for the transmission of CTn214 is nearly identical to a common conjugative transposon found in the genome of *B. fragilis* (CTnDOT), but the autonomously amplified nature of a circular 17,044-nt region of CTn214 that codes for *tetQ* and the integration of the same sequence in the linear chromosome within the same cell is a novel observation. Genome and transcriptome sequencing of *B. fragilis* cultivars grown under different concentrations of tetracycline and ciprofloxacin indicates that *tetQ* in strains containing the circular form remains actively expressed regardless of treatment, while the expression of *tetQ* in strains containing the linear form increases only in the presence of tetracycline.

**IMPORTANCE:**

The exchange of antibiotic production and resistance genes between microorganisms can lead to the emergence of new pathogens. In this study, short-read mapping of metagenomic samples taken over time from the illeal pouch of a patient with ulcerative colitis to a *Bacteroides fragilis* metagenome-assembled genome revealed two distinct genomic arrangements of a novel conjugative transposon, CTn214, that encodes tetracycline resistance. The autonomous amplification of a plasmid-like circular form from CTn214 that includes *tetQ* potentially provides consistent ribosome protection against tetracycline. This mode of antibiotic resistance offers a novel mechanism for understanding the emergence of pathobionts like *B. fragilis* and their persistence for extended periods of time in patients with inflammatory bowel disease.

## INTRODUCTION

Novel functions in a microorganism often arise through conjugation mechanisms that mediate the horizontal transfer of genetic material between co-occurring microbes. Functional conjugative transposons (CTns) have the capacity to shuttle genetic information from a donor cell to a recipient under the control of genes for excision and conjugation necessary for insertion into chromosomal DNA (1, 2). Once the genes are incorporated, the progeny of the transconjugant cell can leverage existing machinery to organize gene regulation and mobilization processes that control flanking or linked genes (3, 4). Conjugative elements commonly carry genes conferring antibiotic protection that allows microbial cells to quickly evolve unique antibiotic resistance strategies (5, 6). Subsequent mutations in these mobile genetic elements sometimes yield new functions that increase fitness (7). Unlike plasmids and phages, which also mediate horizontal gene transfer, CTn elements only transiently exist as extrachromosomal elements during rolling circle replication (8).

Most known CTns extend >60 kbp and carry antibiotic resistance genes (1, 9). Multiple studies have characterized CTn transfer among *Bacteroidetes* genomes. The well-studied CTnDOT occurs in 80% of *Bacteroides* natural isolates where the tetracycline-induced tyrosine site-specific recombinase stimulates excision and transfer (10–12). Tetracycline also stimulates the transcription of the *tetQ* gene, which renders ribosomes tetracycline-resistant and stimulates the regulatory gene *rteA* that activates *rteB,* which triggers the regulatory protein RteC to activate several genes in the excision operon (13, 14). Following excision from the genome, the CTnDOT forms a closed intermediate circular form that is subsequently nicked at the origin of transfer, *oriT*, and then replicated as a double-stranded molecule prior to integrating into the donor or recipient chromosome.

Similar *tetQ* genes occur in the CTns of some *Bacteroides*, *Porphyromonas*, and *Prevotella* species (15–17). Host inflammatory immune response can enhance the horizontal movement of these elements (18) including the transfer of *tetQ* between tetracycline-resistant clinical isolates of *Prevotella*, *Porphyromonas*, and other bacteria (19–21). Comparisons of *tetQ* genes within *Bacteroides* or *Prevotella* have reported high levels of DNA sequence conservation, while comparisons between *tetQ* genes of *Bacteroides fragilis* and *Prevotella intermedia* revealed 99.7% identity (22). Finally, hybrid CTns of diverse origins commonly occur among *Bacteroides* (23, 24).

Many CTns are capable of autonomous rolling circle amplification, which is an important step during the horizontal gene transfer of these elements (25, 26). However, the circular form only exists for a short period of time before the integration into the linear chromosome. In a previous study, we identified a region of a *B. fragilis* genome that contained genes indicative of a conjugative element. Read mapping of the metagenomes from the Human Microbiome Project Data Archive (27) and patients with a history of ulcerative colitis revealed a previously unrecognized genomic architecture and high coverage of a plasmid or plasmid-like region contained within CTn214 (27).

Here, we describe a recently identified conjugative transposon, CTn214, that we initially identified in a *B. fragilis* metagenome-assembled genome (MAG) recovered from longitudinal samples from an inflammatory bowel disease patient with an ileal pouch (27). The cultivation of *Bacteroides* isolates and metagenomic analyses of samples collected from such pouchitis patients during inflamed and normal states offer a means to address several unresolved questions: (i) Which CTn214 genes autonomously amplify? (ii) Can horizontal transfer of CTn214 to other *Bacteroidetes* occur within the same patient following inflammation? (iii) What are the common features shared between this CTn214 and CTns in other *Bacteroides* and *Prevotella* genomes?

## MATERIALS AND METHODS

### Metagenomic-assembled genome recovery

We recovered MAGs from each independent metagenomic sample derived from the patient (p214) harboring the *Bacteroides fragilis* strain that carries CTn214 during both normal and inflamed states. For each sample, we assembled the metagenomic reads using SPAdes v3.14.1 (28). Anvi’o (29, 30) generated a contigs database for each assembly using “anvi-gen-contigs-database”. Bowtie2 v2.4.5 (31) yielded an index of the assembly and reciprocal mapping. Samtools v1.12 (32) filtered and converted the sam files to bam files. Bam files served as input to generate sample profile databases, which were merged into a single database using “anvi-merge.” Anvi’o binned contigs using tetranucleotide similarity, metagenome read coverage, and the presence of single-copy genes. Anvi’o estimated MAG completion and redundancy according to a collection of single-copy genes using HMMER (33) through “anvi-run-hmms.” We searched the single-copy genes in each MAG against the genomes contained in the Genome Taxonomy Database (34) and estimated taxonomy using “anvi-estimate-scg-taxonomy.”

### Characterization of CTn214

Rapid annotations using subsystem technology (RAST) (35) annotated the genes in CTn214, and we searched the MAGs for six genes that encode 15 key protein domains of CTn214, including integrase, DNA topoisomerase III, DNA methylase, TetQ, rteB, and rteC, using hidden Markov models (HMMs) (Table S1). HMMs from the Pfam database (36) were merged into an Anvi’o-compatible database with a noise cutoff term of 1*e*^−25^. We applied these HMM models to the MAGs reconstructed from the metagenomic samples collected from p214 and a collection of cultivars previously reported in reference 29. The cultivar assemblies were derived from four additional patients and included samples collected during inflamed and uninflamed visits (Table S2). Each contigs database was queried for the presence of the 15 domains using “anvi-run-hmms,” and their corresponding sequences were recovered via “anvi-get-sequences-for-hmm-hits.” The frequency of all 15 domains was tabulated into a count matrix using “anvi-script-gen-hmm-hits-matrix-across-genomes” and converted to a presence/absence matrix. We recruited short reads from metagenomic and cultivar samples to genomes and MAGs containing hits to all 15 targets using Bowtie2 v2.4.5 (31). An analysis of variance compared the average fold coverage of the target genes with single-copy genes and multi-operon ribosomal RNA genes. Finally, we aligned CTn214, contigs of MAGs containing each of the 15 target genes within a 70-kbp window, and a contig with 99% sequence identity to CTn214 derived from *Alloprevotella tannerae* ATCC51259. ProgressiveMAUVE aligned sequences using full alignment, default seed weight, iterative refinement, and sum of pair scoring (37).

### CTn214 structure variation

Polymerase chain reaction (PCR) amplifications targeted three distinct regions of CTn214 using DNA from metagenomic samples and cultivars from patient 214. The first amplifies a short fragment of DNA between coding regions for a hypothetical protein and the integrase protein in circular constructs of the 17,044-nt putative autonomous amplicon containing *tetQ*. The second and third assays aimed at the left and right flanking regions of an 11-gene operon inserted between conjugation genes *traG* and *traH*, with expected sizes of ~600 bp and ~350 bp, respectively. PCR reactions employed the Invitrogen Platinum Taq DNA high fidelity polymerase according to the manufacturer’s recommendations with annealing temperatures of 57°C, 61°C, and 57°C for the three distinct regions and 30 cycles of denaturation, annealing, and extension with a final 1-minute 70°C incubation. We visualized PCR fragments on a 1.5% agarose gel and sequenced products on an ABI 3730 capillary sequencer to confirm that sequences matched the expected region of CTn214.

### Antibiotic cultivar treatment

Frozen stocks of two *Bacteroides* strains isolated from patient 214 collected during inflamed and uninflamed visits, visit 7 (genome “p214_V7GG”—Table S2) and visit 8 (genome “p214_V8GG_col1_contigs”—Table S2), respectively, were streaked on *Bacteroides* bile esculin agar plates and incubated at 37°C for 48 hours in an 80% N_2_ and 20% CO_2_ atmosphere using a Coy Laboratory Products anaerobic chamber. Individual colonies from each visit served as inoculants into 1.5 mL of supplemented brain–heart infusion broth followed by incubation for 10 hours at 37°C under anaerobic conditions. Each strain was grown under six different antibiotic culture conditions including 0.5, 1, and 1.5 µg/mL of tetracycline and 1.0, 2.0, and 4.0 µg/mL of ciprofloxacin. After 18 hours of anaerobic growth in 10 mL of brain–heart infusion in the presence of antibiotics, 10 mL of RNAlater (ThermoFisher) was added. Cells were pelleted in a tabletop swinging bucket centrifuge at 3,000 × *g* for 5 minutes, resuspended in 1 mL of supernatant, and transferred to smaller 2-mL tubes. These were spun at 15,000 × *g* for 5 minutes, washed with 1-mL water (HyClone molecular biology grade water; ThermoFisher), and spun again at 15,000 × *g* for 5 minutes. The supernatant was removed, the cell mass was weighed, and it was then stored at −80°C until DNA and RNA extraction. For nucleic acid extractions, the cell pellet was split in an equal ratio for DNA and RNA extractions. DNA was extracted using the DNeasy PowerSoil Kit (Qiagen) according to the manufacturer’s instructions. RNA was extracted using the RNeasy PowerMicrobiome Kit (Qiagen) according to the manufacturer’s instructions. RNA and DNA nucleic acid preparations were resuspended in a 100-μL eluent.

We prepared DNA and RNAseq libraries for each of the unique 24 cultivar–antibiotic treatment experiments outlined above. DNA libraries were prepared using a PCR-free TruSeq Illumina library preparation kit, and RNAseq libraries were prepared using a Nugen prokaryotic preparation kit for RNA. Each DNA and RNA library was uniquely barcoded and sequenced on an Illumina NextSeq platform using a high-throughput 300-cycle kit to produce paired-end 2 × 150-bp reads. Sequencing reads were filtered using Illumina utilities (31, 38). Quality filtered reads were mapped to the CTn214 recovered from the “p214_V8GG_col1_contigs” genome using Bowtie2 v2.2.9 (31). We examined the transcriptome read recruitment across the full length of CTn214 for evidence of high coverage observed in the cultivar and metagenome sequencing.

## RESULTS

### MAG recovery

We recovered 41 MAGs with completion estimates greater than 80% and redundancy less than 10% from six independent assemblies. Each assembly contained a *Bacteroides fragilis* MAG. Five of the six *B. fragilis* MAGs were 5.1 Mbp long and contained 98% of queried single-copy genes, and fewer than 3% of these genes were redundant. The sixth *B. fragilis* MAG was 94% complete and 8% redundant with a length of 4.7 Mbp. This genome was recovered from the earliest available pouch sampling of patient 214, visit 5 (5 M) (Table S2). The taxonomic classification of the remaining MAGs was predominantly *Firmicutes*, with genus-level classifications including *Blautia*, *Clostridium*, *Faecalimonas*, *Ruminocococcus*, and *Enterocloster* (Table S2).

### CTn214 detection and coverage

**Fig 1:**
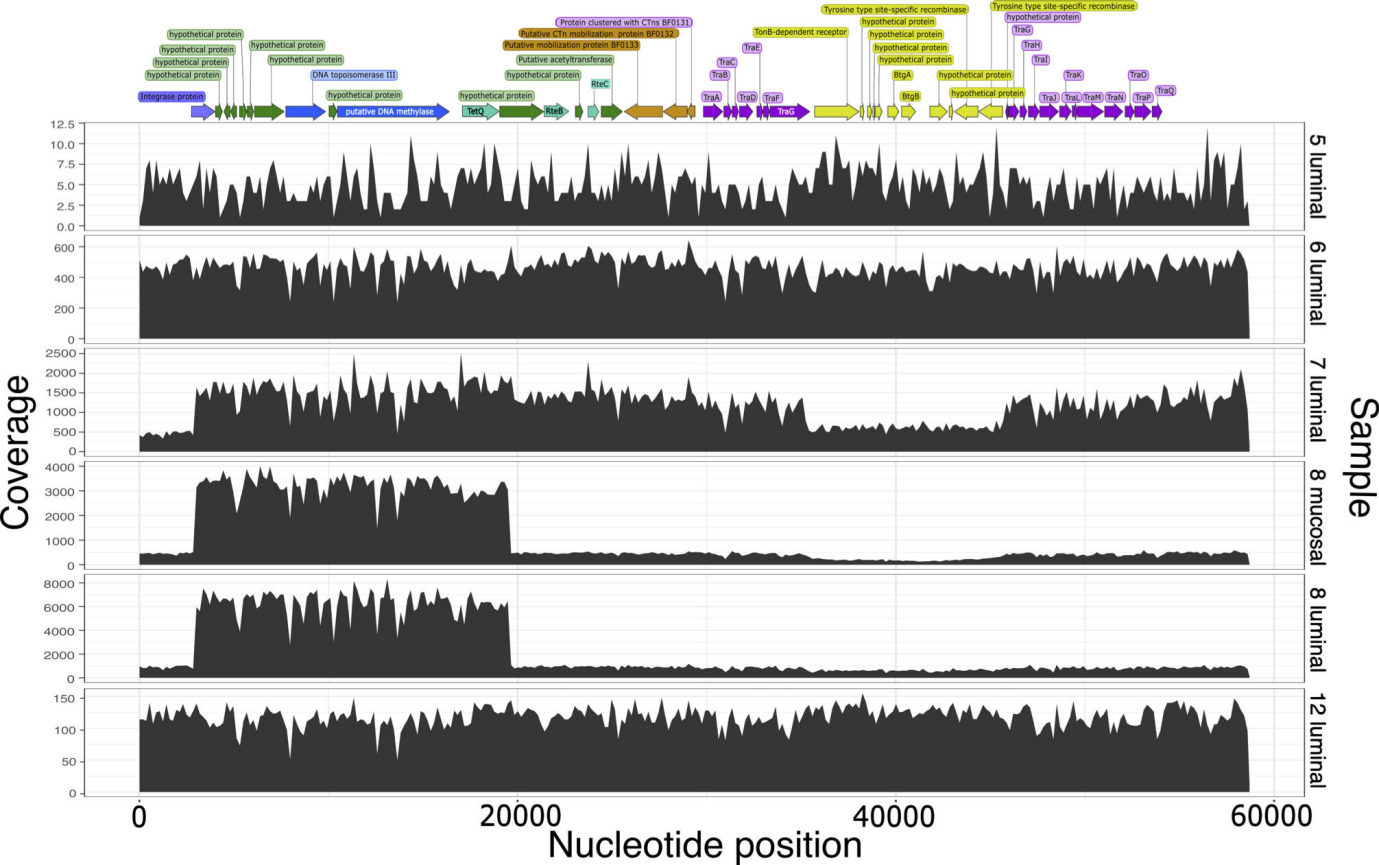
*Bacteroides fragilis* RAST annotation of CTn214 linear form and coverage profiles of short read mapping to CTn214. CTn214 begins with the purple integrase protein, followed by several green hypothetical proteins; the blue annotations are the putative excision operon, followed by the turquoise genes involved in excitation, orange genes for mobilization, purple conjugation genes (TraA-TraG), yellow BtgA and BtgB plasmid mobilization genes, and purple conjugation genes (TraG-TraQ). The black histogram of coverage for each base is displayed for each sample from patient 214. The earliest visit is shown at the top “5 luminal,” and subsequent visits of the longitudinal sampling are plotted below. The date since pouch activation corresponding to the longitudinal sample number is found in Table S2. The average coverage of each nucleotide position of the 55-kbp conjugative transposon is displayed on the left.

We searched the 41 MAGs recovered from six independent metagenomic assemblies from patient 214 in this study and genomes for 13 *Bacteroides* cultivars derived from five other patients (27) (Table S2) for 15 domains that would identify CTn214 and other mobile elements with similar autonomous amplified regions associated with conjugative transposons (Table S2; Fig. 1). We detected all 15 CTn214 domains queried against the six *Bacteroides fragilis* MAGs and six cultivar genomes recovered from patient 214 (Table S2). The *B. fragilis* cultivar from patient 216 also contained hits to the 15 domains, but they were distributed on multiple scaffolds. All other cultivars contained 14 or fewer matches, and *B. fragilis* cultivated from patients 207, 215, 216, and 219 were all missing Toprim_Crpt and rteC (Table S2). MAGs with taxonomic classification other than *Bacteroides* contained fewer than 13 of the 15 CTn214 domains. Genes commonly missing from the incomplete CTn homologs included Toprim_Crpt, rteC, HTH_8, and Phage_int_SAM_5 (Table S2). Genes encoding protein domains classified as Phage_integrase, Phage_int_SAM_5, Response_reg, Sigma54_activate, and GTP_EFTU in the Pfam database were detected at multiple locations in many of the MAGs and cultivars. Response_reg was detected more than 30 times, and we identified more than 100 hits to this target in a single MAG (Table S1). The other domain targets were rarely detected more than 10 times (Table S1).

**Fig 2:**
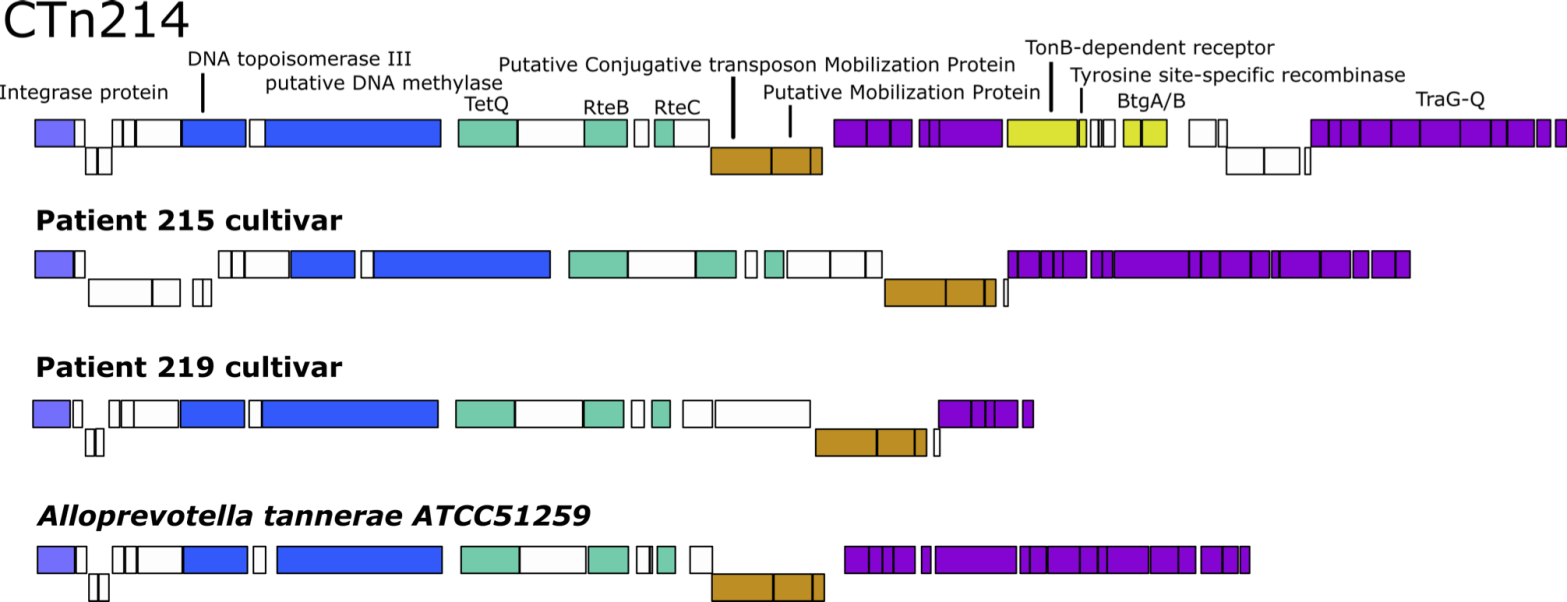
MAUVE alignment and gene annotation of CTn214, *B. fragilis* conjugative transposons derived from cultivars, and *Alloprevotella tannerae* cultivar. Genes are shown as different color blocks, and the color of the block corresponds to the categories of genes outlined in Fig. 1.

The contigs sharing similar gene composition with CTn214 based on the presence of the 15 marker genes indicates that the order of genes beginning with the integrase protein and ending with the *traA-Q* is conserved (Fig. 2). The most notable differences are the genes interrupting the *traA-Q* genes in CTn214 that are not detected in the p215, p219, or the *Alloprevotella tannerae* ATCC51259 strain including *btgA/B* (Fig. 2). Assuming a complete assembly for the conjugative transposon, the cultivar from patient 219 only contained *traA-E*.

A basic local alignment search tool (38) search of the 55-kbp CTn214 against the nonredundant reference sequence database (39) found significant hits to recently sequenced *Bacteroides thetaiotaomicron* cultivar strains BFG-55 (CP081898.1) and BFG-478 (CP103268.1). These two references had 93% query coverage with 96.66% and 94.23% identity, respectively. CTn214 also matched *Alloprevotella* (*Prevotella*) *tannerae* ATCC51259 (NZ_GG700643.1) at 99.97% identity, with 76% query coverage of CTn214.

**Fig 3:**
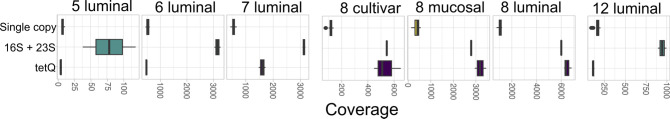
The average read coverage of single copy, 16S and 23S, and the *tetQ* genes are compared using box whisker plots for longitudinal samples collected from patient 214. The timepoint and source (luminal or mucosal) are displayed above each plot.

The metagenomic read mapping to the CTn214 from longitudinal samples reveals striking coverage patterns (Fig. 1). During visits 5 and 6, the average coverage appears to be stable across the entire length of CTn214 (Fig. 1). At visit 7, which preceded inflammation, there are regions of elevated coverage compared to the 5′ end and a 10-kbp portion between conjugative transposon genes *traG* and *traH*. During inflammation at timepoint 8, we observed a sixfold higher coverage relative to single-copy genes for the region containing the integrase protein to *tetQ* in the metagenome of luminal and mucosal samples and the cultivar genome (Fig. 3). There are six ribosomal RNA operons in *B. fragilis* (39), and the coverage of *tetQ* is consistent with the presence of six operons (Fig. 3). The *tetQ* coverage in all other longitudinal samples was comparable to single-copy genes (Fig. 3)

### Chromosomal integrated form *vs.* extrachromosomal circular CTn214

**Fig 4:**
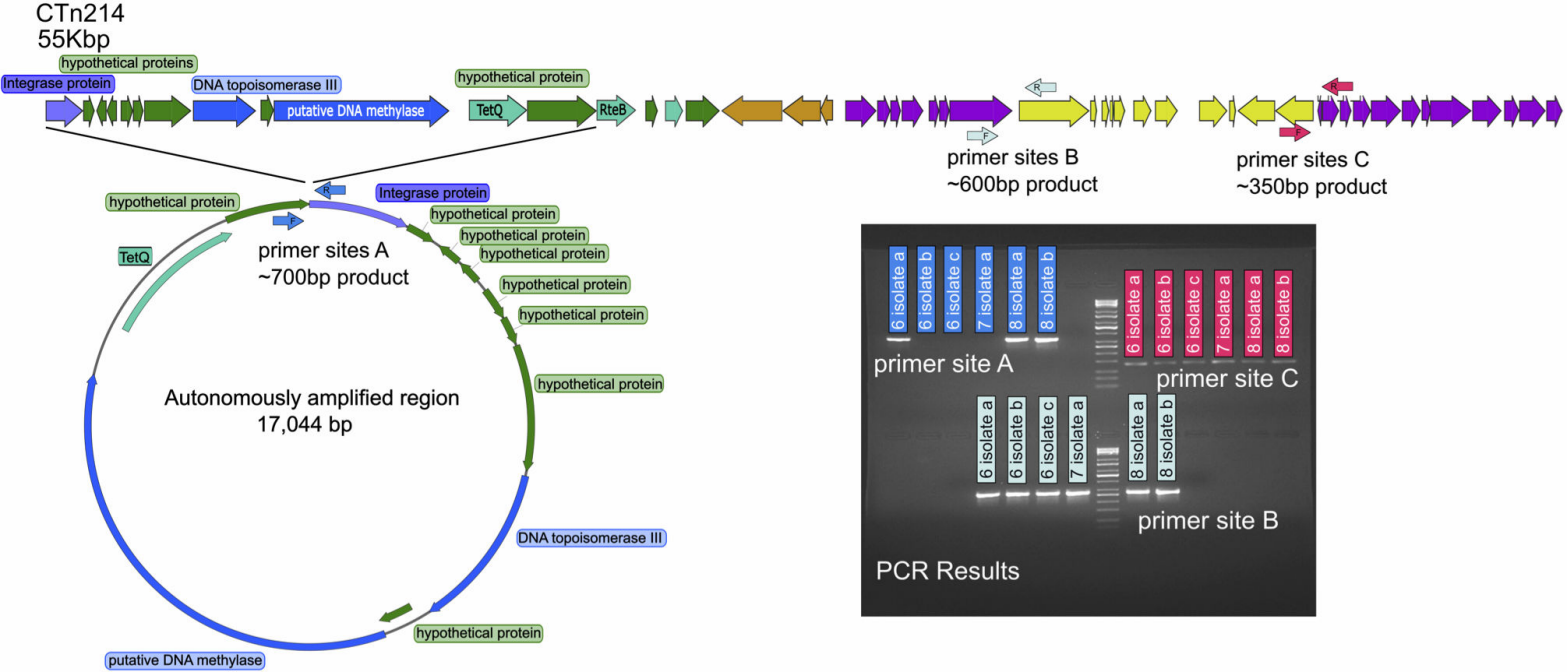
Three regions of the CTn (**A, B, C**) that are targeted for DNA sequence confirmation. (**A**) The primers are designed to amplify a short fragment of DNA that spans the gap between the TetQ and integrase genes that exists only if the region is circularized. (**B**) Primer design to confirm the left flanking region of the clindamycin/tyrosine site-specific recombinase gene insert into the CTn machinery. (**C**) Primer design to confirm the right flanking region of the clindamycin/tyrosine site-specific recombinase gene insert into the CTn machinery.

We observed two distinct coverage profiles of CTn214 that are the potential result of different genomic events. The first coverage profile presents a high coverage of genes spanning an integrase protein through *tetQ* in visit 8 (Fig. 1). This profile could be the result of intrachromosomal gene duplication or extrachromosomal amplification. The second coverage profile that represents a potential genomic event is located around the relatively lower coverage of the 10-kbp element between genes encoding conjugative proteins *traG* and *traH*, beginning with the tonB-dependent receptor and ending with the tyrosine type site-specific recombinase (Fig. 1). This lower coverage region could result from a lower abundance subset of the *B. fragilis* population containing an insertion of these genes or a deletion of the region in the more abundant population. We designed three primer pairs to address the possibility of extrachromosomal amplification vs. intrachromosomal gene duplication and the insertion/deletion of the genomic regions with spurious coverage. Primers were designed to confirm the structures of two unique regions of interest in CTn214*,* described above including a putative autonomously amplified 17,044-nt region and a 10-kbp region inserted within the conjugative machinery between *traG* and *traH*. The primers targeting the putative autonomously amplified circular form of CTn214 produced the expected fragment for three of the *B. fragilis* cultivars from patient 214 (Fig. 4). The *B. fragilis* isolates include three from visit 6, one from visit 7, and two from visit 8. We also detected this circularized fragment in the metagenomic sample from patient 214 visit 8, but all other metagenomic samples were negative (Table S2). Amplification of the flanking regions at the 5′ and 3′ ends of the 10-kbp insert between conjugative transposons, *traG* and *traH* (regions “B” and “C”), produced bands of the predicted size for all cultured isolates from patient 214 (Fig. 4). All other metagenomic samples were negative for region “B” (Table S2), but many produced an appropriately sized product for adjacent genes required for conjugation *traG* and *traH* (Table S2). Examination of the capillary sequences confirmed that the amplicons were produced from the three target regions of CTn214.

### Response to antibiotic treatment

**Fig 5:**
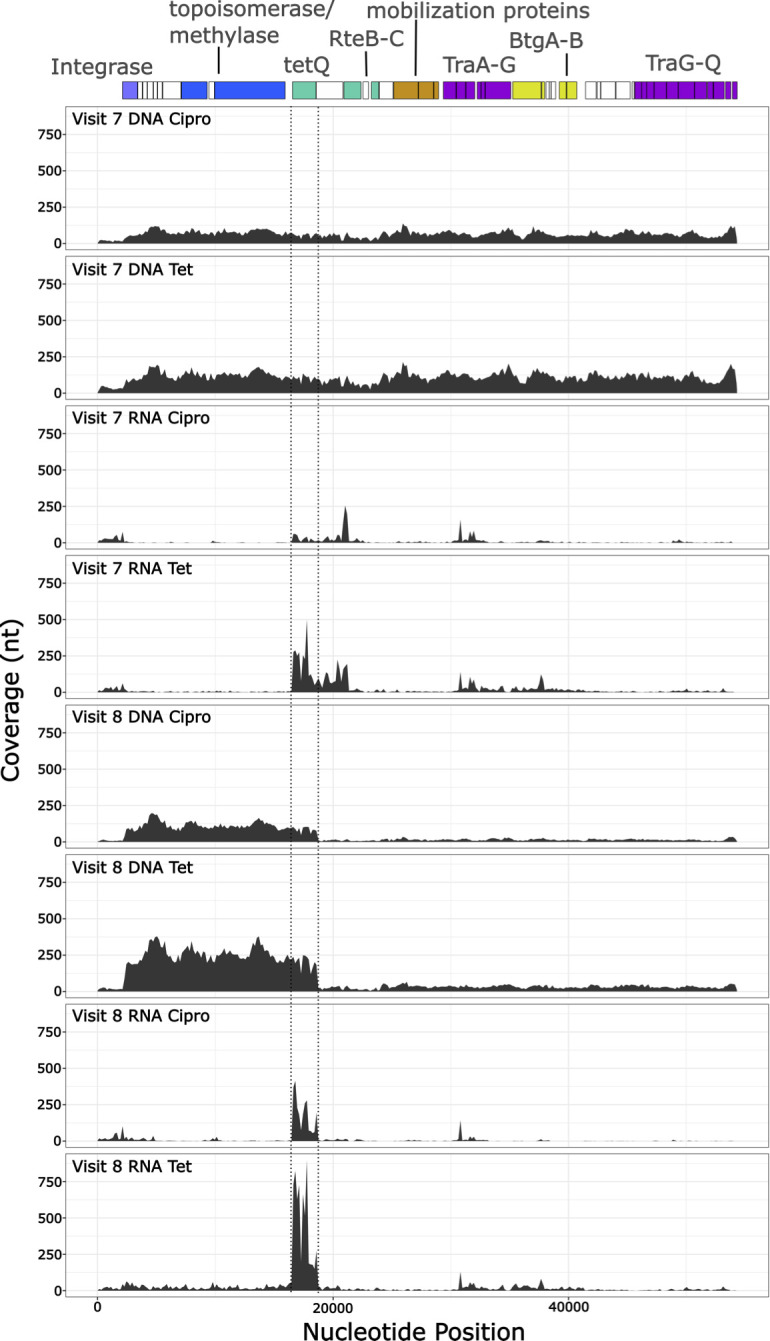
Genomic and transcriptomic sequencing results for two separate cultivars isolated from patient 214, grown in the presence of tetracycline. The annotation of CTn214 is provided at the top of the figure. The panels below the annotation describe the genomic (DNA) or transcriptomic (RNA) average coverage of each nucleotide position of the CTn214 recovered from patient 214 visit 8. The coverage of *tetQ* is shown between the two dotted vertical lines. The coverage displayed was calculated by mapping reads to the visit 8 CTn214 (genome—p214_V8GG_col2_contigs) for all tetracycline experiments, regardless of concentration.

Relative to other chromosomal genes, we found higher genomic (DNA) coverage of the putative autonomously amplified 17,044-nt circular form in the *B. fragilis* isolated from patient 214, visit 8, when cultivated with tetracycline and ciprofloxacin (Fig. 5). We also observed high expression (RNA) of *tetQ* in the strain recovered from visit 8 when grown in the presence of either antibiotic. Conversely, DNA coverage of the 17,044-nt circular form was not above background in the isolate cultivated from visit 7 when grown in the presence of either antibiotic (Fig. 5). The expression of *tetQ* was only notably higher in the cultivar isolated from visit 7 when grown in the presence of tetracycline but not when grown with ciprofloxacin (Fig. 5). We also observed a higher expression of a hypothetical protein adjacent to *tetQ* in visit 7, but the increased expression of this gene did not occur in the visit 8 cultivar. The gene encodes an ATP-binding domain.

## DISCUSSION

The examination of the short-read mapping of metagenomic samples from patient 214 to CTn214 revealed two distinct genomic arrangements of this novel conjugative transposon. The first arrangement is a linear form integrated into the chromosome. The second form includes the linear integrated form and six copies of a 17,044-nt autonomously amplified region of CTn214 carrying the tetracycline resistance gene, *tetQ*. CTn214 contains many of the same genes found in CTnDOT, suggesting that the excitation, excision, and transfer to other *Bacteroidetes* likely occur through the same coordinated series of gene activity stimulated by the presence of tetracycline (11). The occurrence of a linear integrated form combined with circularization and amplification of a subregion renders CTn214 a novel conjugative transposon. *Bacteroides fragilis* isolates recovered from the same patient, at the same timepoint, contain either the linear integrated form alone or the integrated form and a multi-copy circular plasmid. The capacity to mobilize as a conjugative transposon and occur as a multi-copy circular element, carrying tetracycline resistance, represents an important development toward understanding the evolution of antibiotic resistance mechanisms.

The entire length of CTn214 is nearly identical to a conjugative transposon found in *Alloprevotella tannerae* ATCC51259 except for an 11-gene CTn214 operon that includes *btgA, btgB*, two tyrosine site-specific recombinase genes, and a TonB-dependent receptor. This 11-gene operon interrupts *traG*, within the 17 *traA-traQ* genes that encode all the necessary proteins for conjugation. The insertion of the 11 genes at this position may interfere with the expected behavior of CTn214, which for CTnDOT includes excitation by tetracycline, excision, circularization, replication, and transfer from a donor to a recipient cell (11). The *btgA* and *btgB* genes of the 11-gene operon are necessary for the transfer of the machinery for clindamycin resistance in plasmid pBFTM10, and *btgA* binds to *oriT* of plasmid pBFTM10, initiating replication (40). It is possible that *btgA* binds to additional inverted repeats located within CTn214, leading to the observed amplified plasmid-like form containing *tetQ*. We found many potential inverted repeat regions that could serve as an *oriT* site for BtgA binding, cleavage, and initiation of complementary strand synthesis. Additional approaches will be required to understand the role of *btgA* in the amplification of the circular form and how it influences the mobilization of CTn214.

The cultivar isolated from visit 8 of patient 214 maintains six copies of the circular 17,044-nt region irrespective of the presence or absence of several different concentrations of tetracycline or ciprofloxacin. However, the *B. fragilis* cultivar isolated from the previous visit (visit 7) contained only the integrated linear form of CTn214 at all antibiotic concentrations. According to our PCR assay, the visit 7 cultivar is negative for the circular autonomously amplified 17,044-nt fragment, but the genome contains an identical CTn214 found in strains recovered from visit 8. Tetracycline elicited an increase in the production of *tetQ*, *rteB*, and *rteC* in the visit 7 strain, but these genes were nearly below detection when grown in the presence of ciprofloxacin. The activity of *tetQ* in the visit 8 strain was high when grown in the presence of either antibiotic, and the expression of *rteB* and r*teC* was low in comparison to the response of the visit 7 strain. We conclude that there must be some other signals leading to the excision and autonomous amplification of the 17,044-nt plasmid-like form that remains stable in the absence of tetracycline. This observation is surprising, because we expect that cells without the plasmid-like form would grow at a faster rate leading to the eventual loss of the plasmid in the absence of tetracycline. Our observation of low *rteB* and *rteC* activity in the presence of the amplified 17,044-nt plasmid-like form indicates that the plasmid or the insertion between *traA-traQ* may prevent CTn214 from behaving like CTnDOT and instead relies on the activity of the plasmid-like form for tetracycline protection. The interruption of the genes required for conjugation (traA-traQ) would slow or inhibit the transfer of CTn214 to the surrounding microbial community, which could explain why CTn214 may be limited to a single patient, undetectable in the HMPDAC (27), and absent from NCBI reference collection. The ability of strains carrying CTn214 to produce *tetQ* on a constant basis may promote fitness, and this represents an interesting aspect of this strain requiring further inquiry.

The plasmid-like form was not detected in any other MAGs derived from our pouchitis samples, which highlights the potential clinical irrelevance of CTn214. However, elements of CTn214 were common in the healthy patients of the Human Microbiome Project Data Coordination Center (27). Indeed, the observed high-copy plasmid form of CTn214 is rare or specific to a single individual, but the diversity of conjugative elements in *Bacteroides* (2, 41) suggests that other forms have not yet been discovered possibly due to assembly artifacts or bias in metagenomic binning. The detection of circularized and amplified fragments of the genome is difficult because only a fraction of the cells may contain these elements, and they could be misinterpreted by genome assemblers and metagenomic binning algorithms as potential contamination. For example, during visit 6 of the longitudinal sampling of patient 214, we did not see any evidence of a potentially amplified fragment of CTn214 using metagenome mapping. However, PCR confirmed that one of the *B. fragilis* cultivars isolated from this visit contained the circular form. Furthermore, the coverage of the CTn214 in the metagenome sample of visit 7 suggests a mixed population where some of the cells contain the 10-kbp fragment that interrupts the conjugative transposon operon and contains genes related to clindamycin resistance activation. The variable coverage of this region might represent chimeric assembled contigs generated from similar, co-existing strains. This suggests that while metagenomics is an important tool for the discovery of these elements, it may lack the sensitivity to detect low abundance strains containing the circular autonomously replicated 17,044-nt region. Our cultivation and PCR tests were critical to resolving the accuracy of the CTn214 in nature. Novel methods for the discovery of these elements represent a potentially important arena that could shed light on additional aspects of genome variants critically important to not only antibiotic resistance but also virulence (41).

These observations are congruent with suggestions that the discovery of mobile elements providing tetracycline resistance is still in its infancy (42, 43). The multiple forms and behavior of the genes contained in CTn214 represent a single new mechanism that we identified in a single human subject. Our inability to detect the 11 genes that comprise CTn214 within the microbiomes of otherwise healthy patients indicates that CTn214 is not very widespread within the human gut, maintains a low relative abundance, or could be more common within inflamed tissues. Inflammation can stimulate population blooms and the frequency of horizontal gene transfer (18, 27). The description of this element is relevant because host inflammation coincided with a bloom of the *Bacteroides* genome containing CTn214. While CTn214 is not directly involved in eliciting a host inflammatory response, *Bacteroides* blooms are well documented during host inflammation, including pouchitis. Alternative genomic arrangements and mechanisms for antibiotic resistance that we identified in this study are important to the understanding of ecology and evolution of blooms during inflammation and their relevance to human health.

## Data Availability

The metagenomic and cultivar sequences are available through the NCBI Sequence Read Archive dbGaP (accession phs000262) and VAMPS hosts 16S sequences (https://vamps.mbl.edu) under project names HMP_200, HMP_202, HMP_204, HMP_207, HMP_208, HMP_209, HMP_210, HMP_211, HMP_ 212, HMP_213, HMP_214, HMP_215, HMP_216, HMP_217, HMP_218, HMP_219, HMP_423, HMP_427, HMP_500, HMP_502, and HMP_503. DNA and RNA sequence data are deposited in BioProject ID PRJNA1027366. The CTn214 sequence has been submitted to GenBank: OR675140.

## SUPPLEMENTAL MATERIAL

Captions mbio.02787-23-s0001.docx Captions for Tables S1 and S2.

Table S1 mbio.02787-23-s0002.csv Details of the genes recovered from the RAST annotation of CTn214 queried against the Pfam database.

Table S2 mbio.02787-23-s0003.csv Summary of the MAG recovery from each of the independent samples from patient 214.

ASM does not own the copyrights to Supplemental Material that may be linked to, or accessed through, an article. The authors have granted ASM a non-exclusive, world-wide license to publish the Supplemental Material files. Please contact the corresponding author directly for reuse.
