# Supplementary material for: A novel conjugative transposon carrying an autonomously amplified plasmid
Source: mBio. 2024 Jan 23;15(2):e02787-23. doi: 10.1128/mbio.02787-23 (PMC10865816; doi:10.1128/mbio.02787-23)
Supplement: Captions — for Tables S1 and S2. [file mbio.02787-23-s0001.docx]

Supplementary Data - Table 1. Details of the genes recovered from the RAST annotation of CTn214 queried against the Pfam database.

Column 1 is the annotation name provided by RAST, column2 are the domains with significant homology to the RAST gene calls in the Pfam database. The following columns show the length of the RAST gene start and stop positions and the corresponding matches to reference sequecnes in the Pfam database followed by the length of the hit, the bit score, e-value, and the Pfam ID.

Supplementary Data - Table 2. Summary of the MAG recovery from each of the independent samples from patient 214.

The table includes the sample name, days past pouch activation, sample type, genome bin, patient ID and whether the genome analyzed is a MAG or cultivar. The columns beginning with the color background summarize the presence or absence of the protein domain sequences queried in each of the genomes. The color of the background corresponds with the CTn214 genes shown in Figures, 1,2,4 and 5. The remaining columns show the number of genes detected in the queried set of HMMs used to search for CTn214, and the taxonomy and completion scores for each of the MAGs and cultivar genomes.
